# Shifting trends in outpatient hand trauma care: a 16-year analysis at a major center in northern Germany

**DOI:** 10.1007/s00402-024-05745-0

**Published:** 2025-01-04

**Authors:** Martynas Tamulevicius, Florian Bucher, Nadjib Dastagir, Doha Obed, Peter M. Vogt, Khaled Dastagir

**Affiliations:** Hannover Medical School, Hanover, Germany

**Keywords:** Hand injuries, Hand emergencies, Hand trauma, Inpatient treatment

## Abstract

**Background:**

Hand injuries are a leading cause of emergency department visits. Recent trends in hand trauma management reflect a shift toward outpatient care, driven by factors such as a shortage of skilled personnel or increasing cost pressures. This study analyzed these trends to propose updated management strategies for hand injuries.

**Materials and methods:**

This retrospective cohort study included 14,414 patients treated at a certified major hand surgical trauma center between 2007 and 2022. Patients were divided into two groups: the earlier cohort (EC, 2007–2014) and the current cohort (CC, 2015–2022). Trends in inpatient and outpatient care, as well as hospitalization durations, were analyzed.

**Results:**

During the study period, approximately one-third of all patients required inpatient treatment, with one-third of hospitalized patients staying at least one week, one-fifth staying two weeks, and one-tenth staying three or more weeks. Inpatient treatment rates decreased annually by 7%, while outpatient care increased by 5.3% annually. A significant shift toward outpatient management was noted for various injuries, including fractures, burns, lacerations, dislocations, complex injuries, and infections. Despite declining hospitalization rates, patients in the CC group had significantly longer hospital stays, reflecting the increasing complexity and severity of cases requiring admission (*p* < 0.001).

**Conclusions:**

This study reveals a growing trend toward outpatient care for hand injuries, reflecting improved efficiency without compromising quality. Although fewer patients are hospitalized, those admitted require more intensive care, highlighting a shift toward ambulatory management for moderately severe cases. These findings emphasize the importance of initial injury management and underscore the need for expanding outpatient hand surgical care to meet growing demand in a rapidly changing healthcare landscape.

**Level of evidence:**

III.

**Supplementary Information:**

The online version contains supplementary material available at 10.1007/s00402-024-05745-0.

## Introduction

Patients who present with hand and wrist trauma in the emergency department account for up to 30% of all emergency visits [1–6]. Over the past decade, there has been a notable shift in the procedures and management of injuries traditionally performed in inpatient settings to outpatient settings. This trend has been particularly prominent with the advancement of technology, surgical techniques, and the increasing popularity of minimally invasive surgical procedures [7, 8]. On the other hand, the increasing shortage of skilled personnel, aging population, and high-cost pressures have also played a very important role in the shift towards outpatient care [9–11]. The coronavirus disease 2019 (COVID-19) pandemic is also one of the many important catalysts for increasing outpatient care delivery and increasing attention to telemedicine [12, 13]. Hand injuries are particularly common among young adults in their prime working years, with even minor injuries potentially disrupting their ability to work. These injuries often lead to extended work absences, threatening career prospects and sometimes resulting in long-term disability, with significant costs for patients and the healthcare system [14–16]. Providing high-quality care, whether inpatient or outpatient, is therefore essential. Over the last decade, numerous studies have emphasized the importance of treating hand injuries in specialized hand trauma centers [16, 17]. This emphasis has led to a surge in patient presentations at these facilities. However, the inpatient treatment capacity has largely remained unchanged or even decreased, necessitating adjustments in the management strategies for some injuries and a shift toward outpatient care [18]. Nonetheless, there is a notable lack of data concerning the specific shifts in management strategies for different types of hand injuries and the resulting healthcare burdens. This study aims to fill this knowledge gap by examining the evolution and trends in treatment strategies for various hand and wrist injuries.

## Materials and methods

### Study design

In this retrospective, cross-sectional, descriptive epidemiological study, we investigated patients who, between January 2007 and December 2022, presented to a Federation of European Societies for Surgery of the Hand (FESSH)-certified major hand trauma and replantation center at a university hospital in northern Germany. The department is the only facility in the region - serving approximately 1.2 million people - treating the most complex hand injuries. Patients presented with one of the following acute hand injuries: superficial lacerations (shallow cuts or wounds that only affect the outermost layers of the skin or that do not penetrate deeper tissues or structures (subcutaneous tissue, muscles, tendons, nerves, etc.), including contusion, abrasion, insect bite, superficial foreign body), deep lacerations (including one of the following: injury to nerves, blood vessels (arteries or/and deep veins), the articular capsule, muscles or tendons), complex hand injuries requiring complex surgical intervention (including open wounds with a combination of at least two of the following injuries: fractures, injury to nerves, blood vessels, articular capsule, muscles or tendons), amputations, acute wrist fractures (excluding non-unions and pseudoarthroses), metacarpal and finger fractures, joint dislocations, acute joint inflammation, sprains and strains, phlegmon of the hand (including paronychia and panaritium), hand and wrist tenosynovitis, acute joint inflammation and burns.

A comparative analysis was performed, focusing on annual patient demographics (e.g., age, sex), injury characteristics and management strategies across different types of injuries. Using descriptive analyses, trends in inpatient/outpatient treatments over time were identified. For additional comparative analysis, we divided the patient cohort into two groups: Group 1 or the earlier cohort (EC), covering the period between January 2007 and December 2014 (8 years), and Group 2 or the current cohort (CC), covering the period between January 2015 and December 2022 (8 years). Patient demographics and management of the injuries were compared between the groups.

### Data source

All data were retrospectively and anonymously collected from the institutional Enterprise Clinical Research Data Warehouse (ECRDW), an electronic repository integrating the hospital’s administrative records, clinical registries, and patient journals. The ECRDW provided comprehensive datasets, including demographics, injury details and treatment metrics (e.g., inpatient/outpatient status, hospital stay duration, interventions).

### Statistical analysis

The values are shown as the means with standard deviations (SD). Categorical variables are reported as numbers and percentages. A value of *P* < 0.05 was considered significant. Pearson’s correlation was used to determine correlations between continuous variables. Dichotomous variables were compared using Pearson’s chi-squared test. Linear regression analysis was used to analyze the significance of changes in the trends of injuries over time. Categorical variables in the graphs are presented with standard error of the mean (SEM).

## Results

### General trends

During the study period, a total of 14,414 patients with the aforementioned hand injuries presented to our emergency department. The majority of patients were adults and male (Table 1). The mean age of all patients was 40.1 years (SD 19.6). Approximately one-third of all patients (33.79%) were hospitalized for treatment of their injuries. Among these hospitalized patients, 11.04% were under 18 years of age. In both adults and patients under 18 years, the majority of hospitalizations were among male patients (*p* < 0.001 and *p* = 0.011, respectively). Approximately one-third of hospitalized patients stayed for one week or longer, one-fifth for at least two weeks, and one-tenth for three or more weeks in both adults and patients under 18 years. No significant differences were observed in the duration of inpatient treatment between adults and patients under 18 years (*p* = 0.859).

**Table 1:** Distribution of injury rates, gender, and inpatient/outpatient treatment between different age groups

	0–9	10–19	20–29	30–39	40–49	50–59	60–69	70–79	80–89	90+	Total
Injuries	656 (4.6%)	1438 (10.0%)	2833 (19.7%)	2517 (17.5%)	2328 (16.1%)	2177 (15.1%)	1286 (8.9%)	742 (5.1%)	375 (2.6%)	63 (0.4%)	14,414 (100%)
Female	231 (25.2%)	494 (34.4%)	987 (34.8%)	881 (35.0%)	822 (35.3%)	775 (35.6%)	432 (33.6%)	305 (41.1%)	172 (45.9%)	41 (65.1%)	5140 (36%)
Male	435 (64.8%)	944 (65.6%)	1846 (65.2%)	1636 (65.0%)	1506 (64.7%)	1401 (64.4%)	854 (66.4%)	437 (58.9%)	203 (54.1%)	22 (34.9%)	9274 (64%)
Outpatient treatment	388 (59.1%)	1034 (71.9%)	2044 (72.1%)	1785 (70.9%)	1567 (71.2%)	1379 (63.3%)	743 (57.8%)	393 (53.0%)	193 (51.5%)	43 (68.3%)	9659 (67.01%)
Inpatient treatment	268 (40.9%)	404 (28.1%)	751 (26.5%)	693 (27.6%)	749 (32.2%)	798 (36.7%)	543 (42.2%)	349 (47.0%)	182 (48.5%)	20 (31.7%)	4755 (32.98%)
≥ 7 days	69 (3.7%)	150 (8.1%)	276 (15.0%)	276 (15.0%)	321 (17.4%)	320 (17.4%)	206 (11.2%)	143 (7.8%)	75 (4.1%)	8 (0.4%)	1844 (37.84%)
≥ 14 days	35 (3.5%)	82 (8.3%)	138 (14.0%)	154 (15.6%)	174 (17.6%)	186 (18.8%)	109 (11.0%)	66 (6.7%)	38 (3.8%)	6 (0.6%)	988 (20.28%)
≥ 21 days	18 (3.5%)	42 (8.1%)	71 (13.6%)	81 (15.5%)	102 (19.6%)	99 (19.0%)	62 (11.9%)	27 (5.2%)	17 (3.3%)	2 (0.4%)	521 (10.69%)

### Trends of inpatient treatment in different age groups

The age group that experienced the highest frequency of hand injuries consisted of working-age adults. Additionally, approximately two-thirds of the injured individuals were assigned male at birth (Table 1). A significant decrease in injuries among males and an increase in injuries among females was observed starting at age 70. Among patients aged 90 and above, females accounted for two-thirds of all injuries (Table 1). Nonadult patients between the ages of 0 and 9 years had the highest hospitalization rates, exceeding the average of 33.8%, with a rate of 40.9%. Similarly, adults aged 50 to 89 years had hospitalization rates of at least 36.7%. Patients aged 40 to 59 years had the longest duration of inpatient treatment, with one in five being hospitalized for at least 21 days (Table 1).

### Trends in inpatient treatment annually

**Fig. 1:**
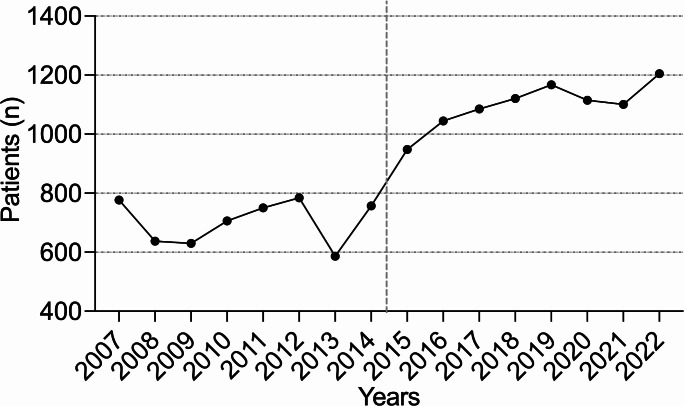
Annual rates of hand injuries

**Fig. 2:**
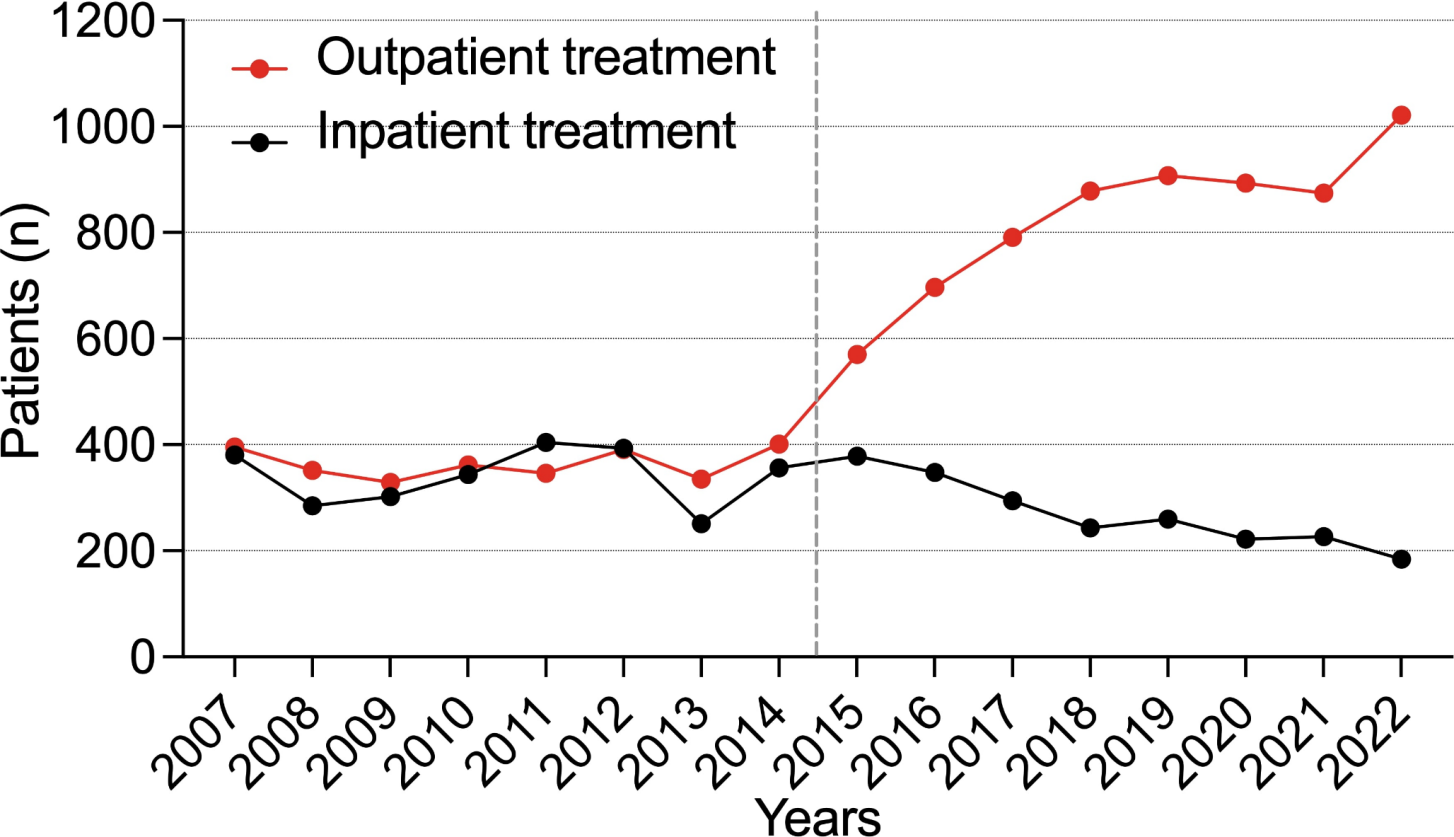
Annual rates of inpatient and outpatient treatments

**Fig. 3:**
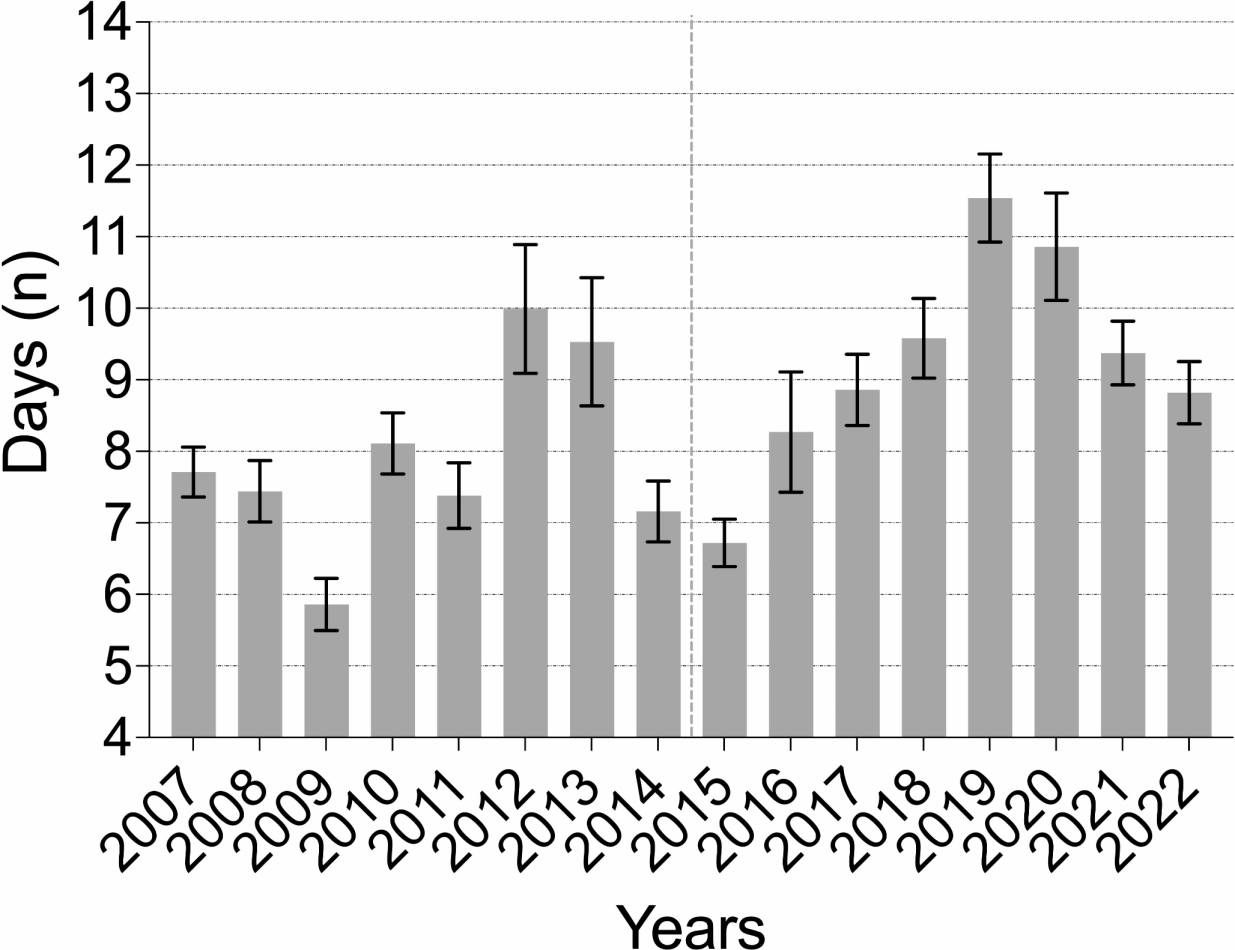
The average duration of hospital stay annually

**Fig. 4:**
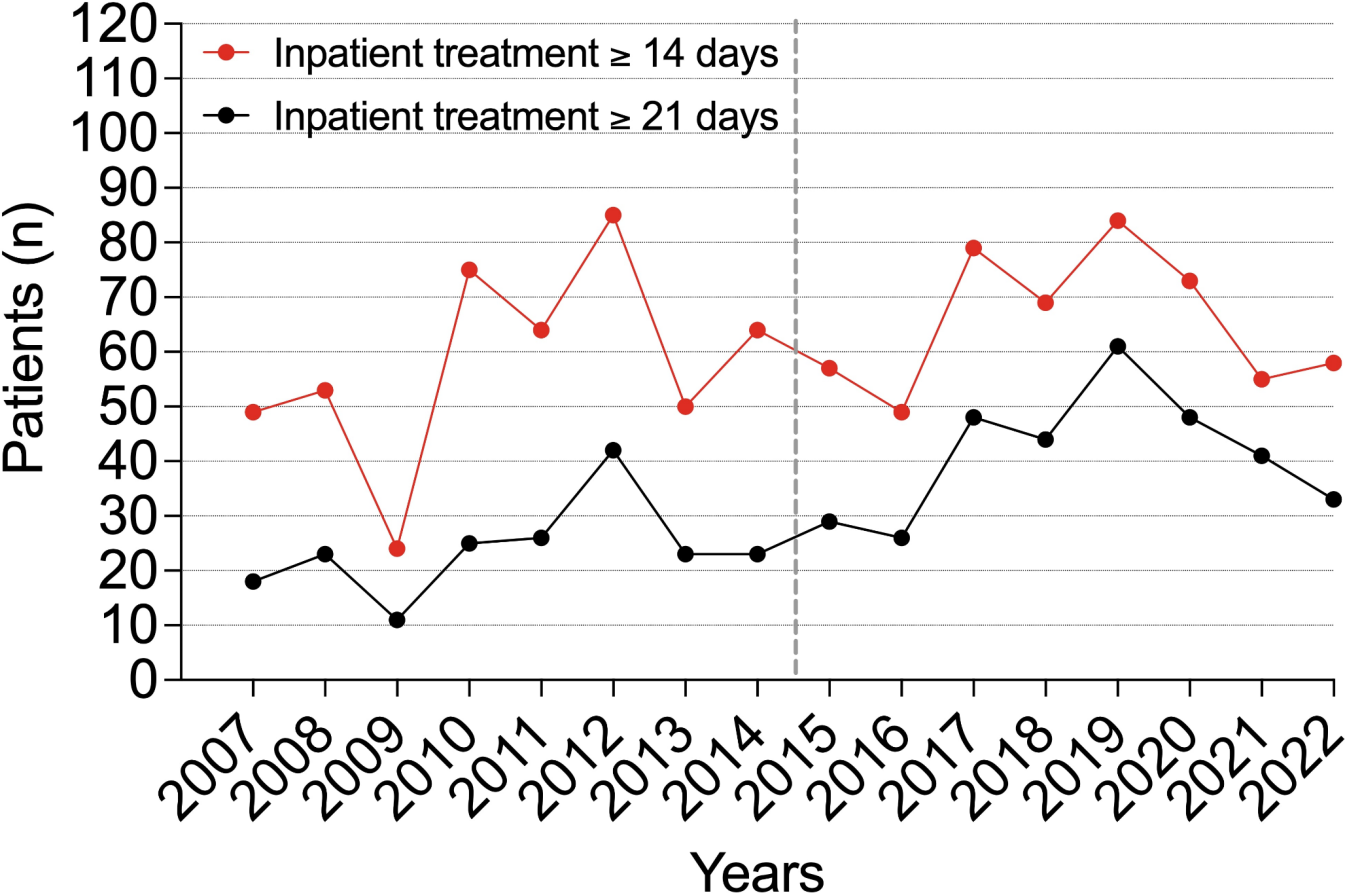
Annual rates of inpatient treatment lasting at least 14 days or 21 days

In the current cohort (CC), the rate of emergency department admissions increased by 56.13%. The number of injuries also showed a steady annual increase (R² = 0.792, *p* < 0.001) (Fig. 1). Throughout the analyzed years, the majority of patients continued to be assigned male at birth. The percentage of patients receiving inpatient treatments decreased significantly by 25.97% (*p* = 0.037), while outpatient treatments increased significantly by 56.08% (*p* < 0.001) (Fig. 2). Inpatient treatments declined at an annual rate of 7% (R² = 0.856, *p* < 0.001), while outpatient treatments rose annually by 5.3% (R² = 0.455, *p* = 0.004). A statistically significant increase in the average duration of hospital stays was observed between the groups (7.79 days (SD 15.28) vs. 9.23 days (SD 19.55), *p* < 0.001) (Fig. 3). Additionally, an annual trend toward longer hospital stays was noted (R² = 0.369, *p* < 0.001). In the EC, almost one in two patients was treated as an inpatient, compared to one in four in the CC. A significant increase in the rate of inpatient treatments lasting 21 days or more was noted (*p* = 0.004), along with an annual increasing trend (R² = 0.507, *p* = 0.002) (Fig. 4).

### Trends in the outpatient treatment of various types of injuries

Further analysis of different injury types revealed a significant shift toward outpatient treatment for deep lacerations, metacarpal and finger fractures, and phlegmon of the hand, with outpatient treatment rates increasing from less than 50% in the earlier cohort (EC) to more than 50% in the current cohort (CC). A significant increase in outpatient treatments was observed for most injury types between the EC and CC groups, except for superficial lacerations (*p* = 0.892), acute wrist fractures (*p* = 0.958), joint dislocations (*p* = 0.490), sprains and strains (*p* = 0.428), hand and wrist tenosynovitis (*p* = 0.872), and acute joint inflammation (*p* = 0.067). A strong significant annual trend was observed for the increase in outpatient treatments for superficial lacerations (R² = 0.774, *p* < 0.001), deep lacerations (R² = 0.819, *p* < 0.001), complex hand injuries (R² = 0.722, *p* < 0.001), metacarpal and finger fractures (R² = 0.822, *p* < 0.001), joint dislocations (R² = 0.682, *p* < 0.001), and phlegmon of the hand (R² = 0.671, *p* < 0.001) (Table S1-12). Additionally, a significant decrease in the duration of hospital stays was noted for complex hand injuries, metacarpal and finger fractures, and joint dislocations (Table 2).

**Table 2:** Annual changes in different types of injuries

Injury type	Injuries in EC (*n*, %)	Injuries in CC (*n*, %)	*P* value	Outpatient treatment in EC (*n*, %*)	Outpatient treatment in CC (*n*, %*)	*P* value	Duration of hospital stay in EC (days)	Duration of hospital stay in CC (days)	*P* value
Superficial lacerations	369 (31.51%)	802 (68.49%)	**< 0.001**	330 (89.43%)	709 (88.40%)	0.892	8.49 (SD 13.27)	7.20 (SD 12.06)	0.444
Deep lacerations	3076 (38.70%)	4881 (61.29%)	**< 0.001**	1331 (43.27%)	3529 (72.30%)	**< 0.001**	5.69 (SD 7.15)	5.17 (SD 8.48)	0.66
Complex hand injuries	1081 (45.04%)	1319 (54.96%)	**< 0.001**	127 (11.74%)	484 (36.69%)	**< 0.001**	6.47 (SD 8.36)	5.36 (SD 8.82)	**0.007**
Amputations	597 (54.97%)	489 (45.03%)	**0.001**	117 (19.60%)	164 (33.54%)	**< 0.001**	8.56 (SD 10.86)	8.11 (SD 9.93)	0.552
Wrist fractures	34 (33.66%)	67 (66.34%)	**0.001**	22 (64.70%)	43 (64.18%)	0.958	12.67 (SD 10.18)	8.61 (SD 6.69)	0.165
Metacarpal and finger fractures	849 (37.82%)	1396 (62.18%)	**< 0.001**	330 (38.87%)	952 (66.26%)	**< 0.001**	5.18 (SD 6.41)	1.89 (SD 3.57)	**0.022**
Joint dislocations	172 (32.76%)	353 (67.24%)	**< 0.001**	64 (37.21%)	164 (46.46%)	0.490	8.94 (SD 11.09)	5.58 (SD 6.15)	**< 0.001**
Sprains and strains	46 (22.77%)	156 (77.23%)	**< 0.001**	43 (93.48%)	140 (89.74%)	0.428	5.67 (SD 3.06)	4.00 (SD 2.33)	0.292
Phlegmon of hand	1112 (46.06%)	1302 (53.94%)	**< 0.001**	475 (42.72%)	734 (56.37%)	**< 0.001**	8.12 (SD 10.76)	7.79 (SD 12.67)	0.625
Hand and wrist tenosynovitis	77 (50.00%)	77 (50.00%)	1.000	34 (44.16%)	36 (46.75%)	0.872	11.58 (SD 9.31)	8.17 (SD 9.44)	0.099
Acute joint inflammations	18 (43.90%)	23 (56.10%)	0.532	1 (5.56%)	6 (26.1%)	0.067	5.82 (SD 4.02)	8.12 (SD 6.75)	0.238
Burns and corrosions	642 (41.37%)	910 (58.63%)	**< 0.001**	410 (63.86%)	661 (72.63%)	**< 0.001**	14.49 (SD 30.69)	11.79 (SD 14.95)	0.215

*Presented as the number and percentage of all injuries in the corresponding cohort (EC or CC)

## Discussion

Our data reveal a significant annual decrease in hospital admissions, declining from approximately 50% in the EC group to 25% in the CC group. By 2022, hospital admissions had dropped further to just 15.3%. This trend aligns with single-center data from some other major European centers [11, 19]. In contrast, a recent 10-year analysis of US emergency departments reported an overall admission rate of just 1.8% [20], similar to rates observed in Belgium and Denmark [1, 19]. The higher admission rates in our study can be attributed to the broad spectrum of injuries included in our analysis. We included 12 distinct groups of hand injuries, encompassing a wide range of conditions, including cases such as hand burns and corrosions. Another contributing factor is the distinct medical insurance policies. The widespread adoption of ambulatory surgery centers in countries like the United States has significantly reduced healthcare costs and facilitated the shift toward outpatient care by offering the same procedures for lower costs for both insurers and patients [21]. In contrast, Germany’s healthcare system, with its universal coverage and differing reimbursement policies, favors hospital-based treatments, as many procedures are not financially attractive for ambulatory centers, which likely contributes to the observed differences in admission rates. Additionally, approximately one-third of hospitalized patients in our cohort required stays of at least one week, and one-tenth were hospitalized for three weeks or more. Longer hospital stays were primarily due to the high complexity of injuries, which often required multiple surgical interventions and could not be managed in outpatient settings. Other contributing factors included patient comorbidities, necessitating additional treatment for collateral conditions, and perioperative complications, particularly wound infections, which required intravenous antibiotic therapy. This trend is partly linked to the aging population in our region and the increasing prevalence of comorbidities among patients [22]. The physiological effects of aging, such as reduced functional reserves, impaired respiratory and cardiovascular responses, and slower healing, complicate the management of older patients, who are also more prone to perioperative complications like cognitive dysfunction, fatigue, and delayed mobilization. These challenges often necessitate extended monitoring in a controlled inpatient environment, as transitioning complex cases to outpatient settings is difficult for this frail population and, in many cases, impossible [23]. Additionally, older adults frequently face inadequate pain management and reluctance to take prescribed analgesics, leading to persistent high pain intensity and delayed recovery [24].

Furthermore, our data indicate a rising overall number of hand injuries treated at our center (R² = 0.792, *p* > 0.001). Notably, the largest increases were observed in minor injuries that could potentially be managed in outpatient settings. The primary reasons for this include the decreasing number of clinics equipped to handle acute hand injuries, an aging workforce in private practices, and inadequate compensation for outpatient procedures in Germany. These factors have made outpatient care less viable for smaller profit-based hospitals and private clinics [22]. An analysis encompassing approximately 1.9 million surgical procedures across various specialties revealed that more than two-thirds (67.8%) of the procedures were conducted on an outpatient basis. Furthermore, discernible patterns toward outpatient surgery were noted, particularly within disciplines such as plastic surgery, including hand surgery, and ophthalmology, where the majority of procedures are performed in outpatient settings [25]. A consensus paper issued by the German Society for Hand Surgery underscores this trend by indicating that approximately 80% of hand surgical procedures can be feasibly conducted on an outpatient basis [26]. Although not specifically addressing hand trauma, this consensus reaffirms that in numerous hand surgery cases, postoperative inpatient care is unnecessary. Carricaburu et al. reported that approximately 70% of traumatic hand injuries were successfully treated in an outpatient surgical setting at a university hand trauma center [19]. Even higher rates were reported by Bhende et al. and Lee et al. from pediatric emergency departments, where approximately 96% of individuals were treated without the need for hospitalization [27, 28]. We observed significantly higher rates of hospitalization for children, as until recently, it has been our routine practice to admit these patients to our clinic for early postoperative care. The average duration of hospital stay in our study cohort was 8.5 days. Many authors do not report the duration of inpatient treatment, especially for different types of injuries. A 10-year analysis of a single hand trauma center in Germany reported slightly lower overall inpatient treatment rates, averaging approximately 5 days [11]. This may be explained by the inclusion of hand burns and corrosions in our analysis, as our clinic serves as a multiregional burn center. Such patients often require longer hospitalization, especially those with deep burns when surgical debridement and wound coverage are necessary.

The distribution of sex and the age group with the most injuries aligned with published data. 64% of patients were men in our study cohort, and these rates are similar to those reported in the Netherlands (62.0%), Denmark (59.0%), Germany (70%) and the United States (60.0%) [3, 11, 20]. The age group that experienced the highest frequency of hand injuries, as well as one of the lowest rates of inpatient treatments, comprised working-age adults between 20 and 59 years. Other authors do not report hospitalization rates, the highest number of injuries was also observed in the same age group [4, 11, 20, 29]. This underscores the concept that hand and wrist injuries frequently impact individuals during their years of active contribution to the workforce, when they are at their peak economic productivity. However, patients aged between 40 and 59 years had the significantly longer duration of inpatient treatment than patients aged 20 and 39 years. This could be explained by the presence of more comorbidities in these patients, leading to a greater risk of postoperative complications, especially those associated with wound healing, such as diabetes or excessive smoking [30–32]. Additionally, individuals in this age group are more likely to operate, or even be employed in occupations that require the use of hazardous equipment, such as saws and other mechanical tools, while remaining active in their daily lives [33]. Such injuries are mostly more complex and require longer inpatient treatment.

Special attention must be given to complex hand injuries and amputations. In both groups, we observed a significant increase in outpatient treatments and a decrease in the duration of hospital stays for admitted patients. This trend may be attributed to improved ambulatory care for hand injuries, enabling the early discharge of patients with more complex hand injuries as well as subtotal amputations after surgical treatment. However, the admission rates for these injuries in our center are notably greater than those reported by authors from the United States. In their analyses of amputations, the indicated admission rates were as low as 20% [34, 35]. Conversely, a recent analysis from a university hospital in Warsaw, Poland, reported almost identical admission rates of approximately 70% [36]. Due to the constraints of this database, we lack the means to determine the factors driving these admission rate trends. We hypothesize that in Europe, complex hand injuries, including amputations, are mostly managed in an inpatient setting at specialized high-volume centers. These centers are inclined to undertake replantation attempts because they have higher success rates [35, 37].

Outpatient surgery has demonstrated significant potential for improving efficiency and reducing costs, with procedures in ambulatory surgery centers taking approximately 25% less time than those performed in hospitals [38]. This increased efficiency, coupled with the ability to meet growing patient demand, highlights the advantages of outpatient care. However, maintaining high treatment standards is crucial. Outpatient surgeries demand the same rigorous hygiene protocols and highly skilled personnel as inpatient settings, particularly as more complex procedures shift to outpatient care. In fact, these transitions often necessitate even higher qualifications among staff to ensure patient safety and optimal outcomes. Research consistently shows that reductions in staff numbers or qualifications can negatively impact the quality of care [39]. Compounding this challenge is a growing shortage of healthcare professionals. By 2020, around 11,000 surgeons in Germany reached retirement age, representing a significant share of both office-based and hospital-based surgeons, and projections suggest that nearly 25% of surgical positions may remain unfilled by 2030 [40]. This workforce shortage poses a considerable barrier to expanding outpatient capacities while maintaining quality standards. Although outpatient care offers clear cost-efficiency advantages, these must not come at the expense of adequately trained personnel or patient outcomes. Investments in workforce development and retention are essential to sustaining the benefits of outpatient surgery without compromising care quality.

This study has several limitations. First, its retrospective design relied on written and coded diagnoses, making the analysis dependent on the accuracy and completeness of medical documentation. While we grouped injuries into broad categories to enhance reliability, this approach inherently oversimplifies some injury subtypes and management variations. Second, the lack of access to complete medical records from the primary care sector limited our ability to analyze comorbidities and medications, which are critical for understanding the factors driving trends in inpatient treatment. Third, we did not analyze patient outcomes, which restricts our ability to assess the long-term effectiveness of the treatment strategies used. Fourth, as a single-center study, our findings may not be generalizable to other regions or institutions, particularly those with different healthcare systems or referral patterns. Future research should explore the associations between comorbidities and specific injuries to better understand how these factors influence treatment strategies for various hand injuries. Expanding the study to include data from multiple high-volume centers would also enhance generalizability and provide insights into regional and systemic variations in care. Moreover, investigating patient outcomes alongside treatment trends and examining the role of smaller clinics and private medical centers in managing minor hand injuries could provide a more comprehensive perspective on the shifting landscape of hand trauma care.

## Conclusions

Our study offers a unique perspective into a broad spectrum of hand injuries, from superficial lacerations to amputations and burns, treated at a university hospital’s hand trauma center. This highlights the considerable burden of hand trauma and illuminates a rising trend toward outpatient treatment and reduced hospital stays across various injuries. Most importantly, while fewer patients overall are hospitalized, those who require admission receive more extensive treatment, suggesting a notable shift toward ambulatory care for moderately severe hand injuries. Nonetheless, our data underscore the crucial role of optimal initial injury management and the increasing need for outpatient hand surgical care.

## Electronic supplementary material

Below is the link to the electronic supplementary material.

Supplementary Material 1

## Data Availability

No datasets were generated or analysed during the current study.
