# Supplementary material for: Shifting trends in outpatient hand trauma care: a 16-year analysis at a major center in northern Germany
Source: Arch Orthop Trauma Surg. 2025 Jan 4;145(1):109. doi: 10.1007/s00402-024-05745-0 (PMC11700033; doi:10.1007/s00402-024-05745-0)
Supplement: Supplementary file 1 — Supplementary Material 1 [file 402_2024_5745_MOESM1_ESM.docx]

Supplementary Table 1. Changes in injury rates, outpatient and inpatient treatment rates, and duration of inpatient treatment for superficial lacerations.

|  | EC | CC | Total | Change between cohorts (%) | P value | R² | P value |
| --- | --- | --- | --- | --- | --- | --- | --- |
| Injuries  (n, %) | 369  (31.51%) | 802  (68.49%) | 1171  (100%) | 117.34 | **<0.001** | 0.788 | **<0.001** |
| Outpatient treatment  (n, %) | 330  (89.43%*) | 709  (88.40%*) | 1039  (88.72%) | 114.84 | 0.892 | 0.027 | 0.377 |
| Inpatient treatment  (n, %) | 39  (10.57%*) | 93  (11.60%*) | 132  (11.27%) | 128.84 |  | 0.183 | 0.098 |
| Duration of inpatient treatment | 8.49 (SD 13.27) | 7.20 (SD 12.06) | 8.01 (SD 12.82) | -11.90 | 0.444 | 0.032 | 0.506 |
| Inpatient treatment  ≥ 7 days  (n, %) | 6  (1.51%*) | 22  (2.74%*) | 28  (21.21%) | 266.67 | 0.657 | 0.098 | 0.238 |
| Inpatient treatment  ≥ 14 days  (n, %) | 3  (0.81%*) | 12  (1.49%*) | 15  (11.36%) | 300.00 | 0.553 | 0.062 | 0.353 |
| Inpatient treatment  ≥ 21 days  (n, %) | 1  (0.27%*) | 8  (0.99%*) | 9  (1.71%) | 700.00 | 0.706 | 0.093 | 0.250 |

**Presented as number and percent of all injuries in corresponding cohort (EC or CC).*

Supplementary Table 2. Changes in injury rates, outpatient and inpatient treatment rates, and duration of inpatient treatment for deep lacerations.

|  | EC | CC | Total | Change between cohorts (%) | P value | R² | P value |
| --- | --- | --- | --- | --- | --- | --- | --- |
| Injuries  (n, %) | 3076  (38.70%) | 4881  (61.29%) | 7948  (100%) | 59.15 | **<0.001** | 0.702 | **<0.001** |
| Outpatient treatment  (n, %) | 1331  (43.27%*) | 3529  (72.30%*) | 4885  (61.46%) | 164.76 | **<0.001** | 0.819 | **<0.001** |
| Inpatient treatment  (n, %) | 1736  (56.73%*) | 1357  (27.70%*) | 3093  (38.54%) | -21.83 |  | 0.369 | **0.013** |
| Duration of inpatient treatment | 5.69 (SD 7.15) | 5.17 (SD 8.48) | 5.46 (SD 7.77) | -10.35 | 0.66 | 0.106 | 0.219 |
| Inpatient treatment  ≥ 7 days  (n, %) | 433  (14.08%*) | 248  (5.82%*) | 681  (8.57%) | -42.73 | **<0.001** | 0.512 | **0.002** |
| Inpatient treatment  ≥ 14 days  (n, %) | 188  (6.11%*) | 104  (2.13%*) | 292  (3.67%) | -44.68 | **<0.001** | 0.223 | 0.061 |
| Inpatient treatment  ≥ 21 days  (n, %) | 55  (1.79%*) | 48  (0.98%*) | 103  (1.29%) | -12.73 | **0.002** | 0.026 | 0.551 |

**Presented as number and percent of all injuries in corresponding cohort (EC or CC).*

Supplementary Table 3. Changes in injury rates, outpatient and inpatient treatment rates, and duration of inpatient treatment for complex hand injuries.

|  | EC | CC | Total | Change between cohorts (%) | P value | R² | P value |
| --- | --- | --- | --- | --- | --- | --- | --- |
| Injuries  (n, %) | 1081  (45.04%) | 1319  (54.96%) | 2400  (100%) | 22.02 | **<0.001** | 0.090 | 0.258 |
| Outpatient treatment  (n, %) | 127  (11.74%*) | 484  (36.69%*) | 611  (25.46%) | 281.10 | **<0.001** | 0.722 | **<0.001** |
| Inpatient treatment  (n, %) | 954  (88.25%*) | 835  (63.31%*) | 1789  (74.54%) | -12.47 |  | 0.176 | 0.106 |
| Duration of inpatient treatment | 6.47 (SD 8.36) | 5.36 (SD 8.82) | 5.95 (SD 8.59) | -17.25 | **0.007** | 0.188 | 0.093 |
| Inpatient treatment  ≥ 7 days  (n, %) | 276  (25.53%*) | 160  (12.13%*) | 436  (18.17%) | -42.03 | **<0.001** | 0.335 | **0.018** |
| Inpatient treatment  ≥ 14 days  (n, %) | 127  (11.75%*) | 69  (5.23%*) | 196  (8.17%) | -45.67 | **<0.001** | 0.148 | 0.141 |
| Inpatient treatment  ≥ 21 days  (n, %) | 41  (3.79%*) | 30  (2.27%*) | 71  (2.96%) | -26.83 | **0.039** | 0.085 | 0.272 |

**Presented as number and percent of all injuries in corresponding cohort (EC or CC).*

Supplementary Table 4. Changes in injury rates, outpatient and inpatient treatment rates, and duration of inpatient treatment for amputations.

|  | EC | CC | Total | Change between cohorts (%) | P value | R² | P value |
| --- | --- | --- | --- | --- | --- | --- | --- |
| Injuries  (n, %) | 597  (54.97%) | 489  (45.03%) | 1086  (100%) | -17.95 | **0.001** | 0.410 | **0.008** |
| Outpatient treatment  (n, %) | 117  (19.60%*) | 164  (33.54%*) | 281  (25.87%) | 40.17 | **<0.001** | 0.241 | 0.053 |
| Inpatient treatment  (n, %) | 480  (80.40%*) | 325  (66.46%*) | 805  (74.13%) | -32.29 |  | 0.650 | **<0.001** |
| Duration of inpatient treatment | 8.56 (SD 10.86) | 8.11 (SD 9.93) | 8.34 (SD 10.49) | -5.72 | 0.552 | 0.048 | 0.416 |
| Inpatient treatment  ≥ 7 days  (n, %) | 196  (32.83%*) | 125  (25.56%*) | 321  (29.55%) | -36.15 | **0.005** | 0.507 | **0.002** |
| Inpatient treatment  ≥ 14 days  (n, %) | 95  (15.91%*) | 58  (11.86%*) | 153  (14.08%) | -38.95 | **0.034** | 0.269 | **0.039** |
| Inpatient treatment  ≥ 21 days  (n, %) | 35  (5.86%*) | 27  (5.52%*) | 62  (5.71%) | -22.86 | 0.896 | 0.096 | 0.243 |

**Presented as number and percent of all injuries in corresponding cohort (EC or CC).*

Supplementary Table 5. Changes in injury rates, outpatient and inpatient treatment rates, and duration of inpatient treatment for wrist fractures.

|  | EC | CC | Total | Change between cohorts (%) | P value | R² | P value |
| --- | --- | --- | --- | --- | --- | --- | --- |
| Injuries  (n, %) | 34  (33.66%) | 67  (66.34%) | 101  (100%) | 97.06 | **0.001** | 0.364 | **0.013** |
| Outpatient treatment  (n, %) | 22  (64.70%*) | 43  (64.18%*) | 65  (64.36%) | 95.45 | 0.958 | 0.314 | **0.023** |
| Inpatient treatment  (n, %) | 12  (35.30%*) | 24  (35.82%*) | 36  (35.64%) | 100.00 |  | 0.125 | 0.178 |
| Duration of inpatient treatment | 12.67 (SD 10.18) | 8.61 (SD 6.69) | 10.00 (SD 8.14) | -5.37 | 0.165 | 0.205 | 0.137 |
| Inpatient treatment  ≥ 7 days  (n, %) | 8  (23.53%*) | 17  (25.37%*) | 25  (24.75%) | 71.43 | 0.839 | 0.122 | 0.205 |
| Inpatient treatment  ≥ 14 days  (n, %) | 6  (17.65%*) | 6  (8.95%*) | 12  (11.88%) | - | 0.213 | 0.001 | 0.877 |
| Inpatient treatment  ≥ 21 days  (n, %) | 3  (8.82%*) | 2  (2.99%*) | 5  (4.95%) | -33.33 | 0.217 | 0.023 | 0.576 |

**Presented as number and percent of all injuries in corresponding cohort (EC or CC).*

Supplementary Table 6. Changes in injury rates, outpatient and inpatient treatment rates, and duration of inpatient treatment for metacarpal and finger fractures.

|  | EC | CC | Total | Change between cohorts (%) | P value | R² | P value |
| --- | --- | --- | --- | --- | --- | --- | --- |
| Injuries  (n, %) | 849  (37.82%) | 1396  (62.18%) | 2245  (100%) | 64.43 | **<0.001** | 0.784 | **<0.001** |
| Outpatient treatment  (n, %) | 330  (38.87%*) | 952  (66.26%*) | 1282  (57.10%) | 188.48 | **<0.001** | 0.822 | **<0.001** |
| Inpatient treatment  (n, %) | 519  (61.13%*) | 444  (33.74%*) | 936  (42.90%) | -14.45 |  | 0.245 | 0.051 |
| Duration of inpatient treatment | 5.18 (SD 6.41) | 1.89 (SD 3.57) | 3.63 (SD 5.05) | -15.61 | **0.022** | 0.173 | 0.109 |
| Inpatient treatment  ≥ 7 days  (n, %) | 175  (20.61%*) | 109  (7.81%*) | 284  (12.65%) | -37.71 | **<0.001** | 0.308 | **0.026** |
| Inpatient treatment  ≥ 14 days  (n, %) | 81  (9.54%*) | 48  (3.43%*) | 129  (5.74%) | -40.74 | **<0.001** | 0.103 | 0.226 |
| Inpatient treatment  ≥ 21 days  (n, %) | 27  (3.18%*) | 21  (1.50%*) | 48  (2.14%) | -22.22 | **0.010** | 0.026 | 0.548 |

**Presented as number and percent of all injuries in corresponding cohort (EC or CC).*

Supplementary Table 7. Changes in injury rates, outpatient and inpatient treatment rates, and duration of inpatient treatment for joint dislocations.

|  | EC | CC | Total | Change between cohorts (%) | P value | R² | P value |
| --- | --- | --- | --- | --- | --- | --- | --- |
| Injuries  (n, %) | 172  (32.76%) | 353  (67.24%) | 525  (100%) | 105.23 | **<0.001** | 0.473 | **0.003** |
| Outpatient treatment  (n, %) | 64  (37.21%*) | 164  (46.46%*) | 228  (43.43%) | 156.25 | 0.49 | 0.682 | **<0.001** |
| Inpatient treatment  (n, %) | 108  (62.79%*) | 189  (53.54%*) | 297  (56.57%) | 75.00 |  | 0.102 | 0.214 |
| Duration of inpatient treatment | 8.94 (SD 11.09) | 5.58 (SD 6.15) | 6.80 (SD 8.43) | -41.27 | **<0.001** | 0.320 | **0.022** |
| Inpatient treatment  ≥ 7 days  (n, %) | 46  (26.74%*) | 46  (13.06%*) | 92  (17.52%) | - | **<0.001** | 0.005 | 0.804 |
| Inpatient treatment  ≥ 14 days  (n, %) | 20  (11.63%*) | 18  (5.11%*) | 38  (7.23%) | -10.00 | **0.011** | 0.006 | 0.762 |
| Inpatient treatment  ≥ 21 days  (n, %) | 9  (5.23%*) | 5  (1.42%*) | 14  (2.67%) | -44.44 | **0.014** | 0.061 | 0.358 |

**Presented as number and percent of all injuries in corresponding cohort (EC or CC).*

Supplementary Table 8. Changes in injury rates, outpatient and inpatient treatment rates, and duration of inpatient treatment for sprains and strains.

|  | EC | CC | Total | Change between cohorts (%) | P value | R² | P value |
| --- | --- | --- | --- | --- | --- | --- | --- |
| Injuries  (n, %) | 46  (22.77%) | 156  (77.23%) | 202  (100%) | 262.79 | **<0.001** | 0.611 | **<0.001** |
| Outpatient treatment  (n, %) | 43  (93.48%*) | 140  (89.74%*) | 183  (90.59%) | 225.58 | 0.428 | 0.548 | **0.001** |
| Inpatient treatment  (n, %) | 3  (6.52%*) | 16  (10.26%*) | 19  (9.41%) | 433.33 |  | 0.178 | 0.103 |
| Duration of inpatient treatment | 5.67 (SD 3.06) | 4.00 (SD 2.33) | 4.26 (SD 2.45) | 120.59 | 0.292 | 0.056 | 0.377 |
| Inpatient treatment  ≥ 7 days  (n, %) | 1  (2.17%*) | 3  (1.92%*) | 4  (1.98%) | 200.00 | 0.915 | 0.119 | 0.191 |
| Inpatient treatment  ≥ 14 days  (n, %) | 0 | 0 | 0 | - | - | - | - |
| Inpatient treatment  ≥ 21 days  (n, %) | 0 | 0 | 0 | - | - | - | - |

**Presented as number and percent of all injuries in corresponding cohort (EC or CC).*

Supplementary Table 9. Changes in injury rates, outpatient and inpatient treatment rates, and duration of inpatient treatment for phlegmon of hand.

|  | EC | CC | Total | Change between cohorts (%) | P value | R² | P value |
| --- | --- | --- | --- | --- | --- | --- | --- |
| Injuries  (n, %) | 1112  (46.06%) | 1302  (53.94%) | 2414  (100%) | 17.09 | **<0.001** | 0.127 | 0.175 |
| Outpatient treatment  (n, %) | 475  (42.72%*) | 734  (56.37%*) | 1209  (50.08%) | 54.53 | **<0.001** | 0.671 | **<0.001** |
| Inpatient treatment  (n, %) | 637  (57.28%*) | 568  (43.63%*) | 1205  (49.92%) | -10.83 |  | 0.109 | 0.211 |
| Duration of inpatient treatment | 8.12 (SD 10.76) | 7.79 (SD 12.67) | 7.97 (SD 11.69) | -4.58 | 0.625 | 0.024 | 0.563 |
| Inpatient treatment  ≥ 7 days  (n, %) | 228  (20.50%*) | 175  (13.44%*) | 403  (16.69%) | -23.25 | **<0.001** | 0.197 | 0.085 |
| Inpatient treatment  ≥ 14 days  (n, %) | 104  (9.35%*) | 74  (56.92%*) | 178  (7.37%) | -28.85 | **<0.001** | 0.088 | 0.263 |
| Inpatient treatment  ≥ 21 days  (n, %) | 43  (3.87%*) | 40  (3.07%*) | 83  (3.44%) | -6.98 | 0.314 | 0.020 | 0.602 |

**Presented as number and percent of all injuries in corresponding cohort (EC or CC).*

Supplementary Table 10. Changes in injury rates, outpatient and inpatient treatment rates, and duration of inpatient treatment for hand and wrist tenosynovitis.

|  | EC | CC | Total | Change between cohorts (%) | P value | R² | P value |
| --- | --- | --- | --- | --- | --- | --- | --- |
| Injuries  (n, %) | 77  (50.00%) | 77  (50.00%) | 154  (100%) | - | 1.000 | 0.023 | 0.574 |
| Outpatient treatment  (n, %) | 34  (44.16%*) | 36  (46.75%*) | 70  (45.45%) | 5.88 | 0.872 | 0.011 | 0.696 |
| Inpatient treatment  (n, %) | 43  (55.84%*) | 41  (53.25%*) | 84  (54.55%) | -4.65 |  | 0.025 | 0.555 |
| Duration of inpatient treatment | 11.58 (SD 9.31) | 8.17 (SD 9.44) | 9.92 (SD 9.47) | -31.34 | 0.099 | 0.254 | **0.046** |
| Inpatient treatment  ≥ 7 days  (n, %) | 21  (27.27%*) | 16  (20.78%*) | 40  (25.97%) | -33.33 | 0.198 | 0.023 | 0.577 |
| Inpatient treatment  ≥ 14 days  (n, %) | 16  (20.78%*) | 5  (6.49%*) | 21  (13.64%) | -68.75 | **0.017** | 0.260 | **0.043** |
| Inpatient treatment  ≥ 21 days  (n, %) | 7  (9.09%*) | 4  (5.19%*) | 11  (7.14%) | -42.86 | 0.533 | 0.034 | 0.492 |

**Presented as number and percent of all injuries in corresponding cohort (EC or CC).*

Supplementary Table 11. Changes in injury rates, outpatient and inpatient treatment rates, and duration of inpatient treatment for acute joint inflammations.

|  | EC | CC | Total | Change between cohorts (%) | P value | R² | P value |
| --- | --- | --- | --- | --- | --- | --- | --- |
| Injuries  (n, %) | 18  (43.90%) | 23  (56.10%) | 41  (100%) | 27.78 | 0.532 | 0.040 | 0,457 |
| Outpatient treatment  (n, %) | 1  (5.56%*) | 6  (26.1%*) | 7  (17.1%) | 500.00 | 0.067 | 0.171 | 0.111 |
| Inpatient treatment  (n, %) | 17  (94.4%*) | 17  (73.9%*) | 34  (82.9%) | 0.00 |  | 0.001 | 0.932 |
| Duration of inpatient treatment | 5.82 (SD 4.02) | 8.12 (SD 6.75) | 6.97 (SD 5.59) | 70.32 | 0.238 | 0.079 | 0.290 |
| Inpatient treatment  ≥ 7 days  (n, %) | 5  (27.78%*) | 7  (30.43%*) | 12  (29.27%) | 40.00 | 0.566 | 0.051 | 0.401 |
| Inpatient treatment  ≥ 14 days  (n, %) | 1  (5.56%*) | 3  (13.04%*) | 4  (9.75%) | 200.00 | 0.410 | 0.063 | 0.349 |
| Inpatient treatment  ≥ 21 days  (n, %) | 0 | 2  (8.69%*) | 2  (4.88%) | - | 0.122 | 0.108 | 0.215 |

**Presented as number and percent of all injuries in corresponding cohort (EC or CC).*

Supplementary Table 12. Changes in injury rates, outpatient and inpatient treatment rates, and duration of inpatient treatment for burns and corrosions.

|  | EC | CC | Total | Change between cohorts (%) | P value | R² | P value |
| --- | --- | --- | --- | --- | --- | --- | --- |
| Injuries  (n, %) | 642  (41.37%) | 910  (58.63%) | 1552  (100%) | 41.74 | **<0.001** | 0.429 | **0.006** |
| Outpatient treatment  (n, %) | 410  (63.86%*) | 661  (72.63%*) | 1071  (69.01%) | 61.22 | **<0.001** | 0.346 | **0.017** |
| Inpatient treatment  (n, %) | 232  (36.14%*) | 249  (27.36%*) | 481  (30.99%) | 7.33 |  | 0.068 | 0.329 |
| Duration of inpatient treatment | 14.49 (SD 30.69) | 11.79 (SD 14.95) | 13.09 (SD 23.89) | -16.35 | 0.215 | 0.082 | 0.283 |
| Inpatient treatment  ≥ 7 days  (n, %) | 128  (19.94%*) | 122  (13.40%*) | 250  (16.11%) | -4.69 | **<0.001** | 0.018 | 0.617 |
| Inpatient treatment  ≥ 14 days  (n, %) | 80  (12.46%*) | 67  (7.36%*) | 147  (9.47%) | -16.25 | **<0.001** | 0.109 | 0.211 |
| Inpatient treatment  ≥ 21 days  (n, %) | 40  (6.23%*) | 42  (4.62%*) | 82  (5.28%) | 5.00 | 0.168 | 0.001 | 0.967 |

**Presented as number and percent of all injuries in corresponding cohort (EC or CC).*
